# Towards non-target proactive food safety: identification of active compounds in convenience tomato products by ten-dimensional hyphenation with integrated simulated gastrointestinal digestion

**DOI:** 10.1007/s00216-023-04656-0

**Published:** 2023-03-29

**Authors:** Tamara Schreiner, Naila M. Eggerstorfer, Gertrud E. Morlock

**Affiliations:** Institute of Nutritional Science, Chair of Food Science, Justus Liebig University Giessen, Heinrich-Buff-Ring 26-32, 35392 Giessen, Germany

**Keywords:** High-performance thin-layer chromatography, Planar bioassay, High-performance liquid chromatography, High-resolution mass spectrometry, Bioactivity through digestion

## Abstract

Current strategies for non-target food screening focus mainly on known hazardous chemicals (adulterants, residues, contaminants, packaging migrants, etc.) instead of bioactive constituents in general and exclude the biological effect detection. To widen the perspective, a more proactive non-target effect-directed strategy is introduced to complement food safety in order to detect not only known but also unknown bioactive compounds. The developed 10-dimensional hyphenation included on-surface digestion (1D), planar chromatographic separation (2D), visualization using white light (3D), UV light (4D), fluorescence light (5D), effect-directed assay analysis (6D), heart-cut zone elution to an orthogonal reversed phase column chromatography including online desalting (7D) with subsequent diode array detection (8D), high-resolution mass spectrometry (9D), and fragmentation (10D). Metabolism, i.e., intestinal digestion of each sample, was simulated and integrated on the same adsorbent surface to study any changes in the compound profiles. As proof of principle, nine convenience tomato products and a freshly prepared tomato soup were screened via five different planar assays in a non-targeted mode. Non-digested and digested samples were compared side by side. In their effect-directed profiles, 14 bioactive compounds from classes of lipids, plant hormones, spices, and pesticides were identified. In particular, bioactive compounds coming from the lipid class were increased by gastrointestinal digestion, while spices and pesticides remained unaffected. With regard to food safety, the determination of the two dinitrophenol herbicides dinoterb and dinoseb in highly processed tomato products should be given special attention. The hyphenation covered a broad analyte spectrum and showed robust and reliable results.

**Graphical Abstract:**

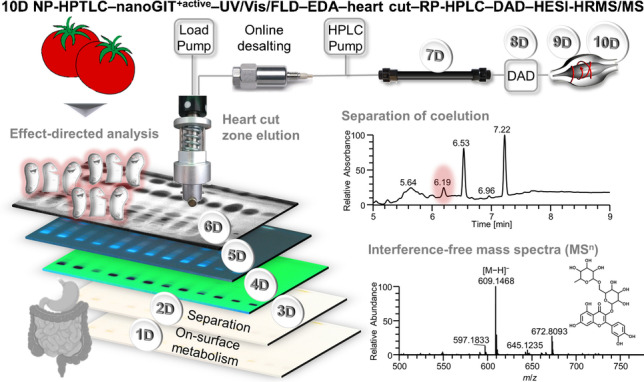

**Supplementary Information:**

The online version contains supplementary material available at 10.1007/s00216-023-04656-0.

## Introduction

The screening for hazardous substances in our daily diet is important to ensure food safety and consumer protection. Non-target screening strategies of food tend to focus on hazardous chemicals from packaging materials [1], antibiotic residues in animal products [2–4], or pesticide residues in fruits and vegetables [5, 6]. Screening for health-promoting compounds and their bioavailability is as important, since beneficial compounds also present in a complex sample could mitigate hazardous effects. However, setting priority criteria, which unknown compound signals from the thousands in complex samples (as given for food or environmental samples, etc.) are worth elucidating, remains challenging [7]. Advantages of using multi-hyphenated non-target strategies for bioactive compound screening in foods were recently described [8–11]. These strategies were proactive in that each active compound that showed an effect was identified. In addition, the combination with bioavailability tools is important since substance (de)activation can be mediated through microbial or enzymatic activity during digestive processes or further metabolism pathways, which may change bioactivity and bioaccessibility [12–14]. The latter is defined by the release of dietary nutrients from the food matrix, solubilization, digestive stability, and also the efficiency of absorption through the gut walls [15, 16]. Bioactivity can be influenced through proteolysis by proteases, split-off of glycosidic sugar moiety by amylases, hydrolysis of acylglycerols to free fatty acids (FFAs) by lipases [16] or toxification/detoxification by liver enzymes [17]. In particular, the focus of new non-target screening strategies should be on digestibly stable bioactive substances from the daily diet and those getting activated by digestion or other metabolic pathways. Both hazardous and beneficial bioactive compounds as well as their metabolic fate (activation/deactivation) need to be known to ensure proactive consumer protection and are worth clarifying their structure.

Static on-surface [17] as well as static in vitro [12, 18–20] digestion models are subdivided into oral, gastric, and intestinal phase [17, 19], trying to mimic in vivo digestion conditions dependent on microflora, pH, and temperature [15]. The enzymatic composition of the internal fluids differs between the three phases. While α-amylase is the predominant enzyme in the oral phase, it is the protease pepsin for the acidic gastric phase and proteolytic, lipolytic, and amylolytic enzymes in the intestinal phase [15, 16]. Since all enzyme classes are present in the intestinal phase and pre-digested food remains longer in the intestine than in mouth and gut, the intestinal phase is a good choice to mimic digestive processes in vitro. Most protocols perform the in vitro simulated digestion prior to chromatographic separation and subsequent biochemical or biological detection to evaluate changes through digestion [21–23]. Status-quo is the separation of a complex mixture by column chromatography into many fractions or substance peaks collected in microtiter plate wells. Subsequently, the bioactivity is determined therein which prohibits the direct assignment of bioactivity and metabolic changes to individual substances because microtiter plate assays show only sum values even after fractionation. Moreover, the analysis time is doubled since all experiments are performed twice, for non-digested as well as digested samples. To overcome these problems, the simulated static digestion was developed on the adsorbent surface and integrated into the planar chromatography workflow [17], allowing effect-directed detection of bioactive compounds and side-by-side comparison of non-digested and digested samples.

In this study, a ten-dimensional (10D) hyphenation strategy for non-targeted and proactive screening of bioactive compounds in food was developed for the first time. The workflow contained a miniaturized intestinal on-surface digestion at the nanomolar compound scale (nanoGIT^+active^) to mimic true-to-life conditions. It allowed through the simulated metabolic processes and effect-directed analysis/detection (EDA) the identification of changes in the bioactive compound profiles of complex samples. Different normal phase high-performance thin-layer chromatography (NP-HPTLC) solvent systems and multi-detection (UV/Vis/FLD), reversed phase high-performance liquid chromatography (RP-HPLC) gradients and diode array detection (DAD), and tandem high-resolution mass spectrometry (HRMS/MS) acquisition methods were studied. The proof-of-concept of the developed NP-HPTLC–nanoGIT^+active^–UV/Vis/FLD–EDA–heart cut–RP-HPLC–DAD–HESI-HRMS/MS method was demonstrated for raw extracts of nine convenience tomato products versus a freshly prepared tomato soup. The effect-directed profiles were compared directly and after intestinal digestion. It was hypothesized that this disruptive strategy will provide a non-target food screening which is more efficient and, in particular, more proactive regarding food safety and consumer protection compared to existing strategies.

## Materials and methods

### Chemicals and materials

Double distilled water was prepared by a Heraeus Destamat Bi-18E from Thermo Fisher Scientific, Dreieich, Germany. All solvents were of chromatography grade, and all salts of *pro analysis* quality unless stated otherwise. Rivastigmine (≥ 98%), sodium acetate (> 99%), peptone from casein (for microbiology), caffeine (reagent plus), imidazole (≥ 99.5%), acarbose (≥ 95%), quercetin (≥ 95%), L-ascorbic acid (reagent grade), naringenin (≥ 95%), myristic acid (≥ 99%), palmitic acid (> 99%), linoleic acid (60–74%), bile extract (porcine), pancreatin from porcine pancreas (8 × USP specifications), acetylcholinesterase (AChE) from *Electrophorus electricus* (≥ 245 U/mg, 10 kU/vial), and α-glucosidase from *Saccharomyces cerevisiae* (1,000 U/vial) were purchased from Sigma-Aldrich, Steinheim, Germany. Ammonium carbonate (extra pure) was delivered by Bernd Kraft, Duisburg, Germany. β-Glucosidase from almonds (3040 U/mg) and 2-naphthyl-β-d-glucopyranoside (95%) were provided by ABCR, Karlsruhe, Germany. Acetonitrile (ACN, ≥ 99.8%, Honeywell, Riedel-de Haën), ammonium acetate (≥ 99%), ammonium formate (LC–MS grade, ≥ 99%), and sodium bicarbonate (≥ 99.7%) were obtained from Fluka, Sigma-Aldrich, Steinheim, Germany. Chlorogenic acid (≥ 95%) was from Cayman Chemical, Ann Arbor, MI, USA. Bovine serum albumin (BSA, fraction V, ≥ 98%), tris(hydroxymethyl)aminomethane (≥ 99.9%), hydrochloric acid (purest, 37%), ethanol, glacial acetic acid, sodium dihydrogen phosphate monohydrate (99%), dipotassium hydrogen phosphate trihydrate (≥ 99%), glycerol (Rotipuran, 86%), stearic acid (> 98%), oleic acid (> 99%), *n*-hexane (≥ 98%), calcium chloride (≥ 98%), potassium dihydrogen phosphate (≥ 99%), magnesium chloride hexahydrate (≥ 98%), and disodium hydrogen phosphate (≥ 99%) were delivered by Carl Roth, Karlsruhe, Germany. 2-Naphtyl-α-d-glucopyranoside (99%) was from Fluorochem, Hadfield Derbyshire, UK. HPTLC silica gel 60 F_254_ MS grade plates, magnesium sulfate heptahydrate (99.5%), and potassium chloride (≥ 99.5%) were provided by Merck, Darmstadt, Germany. Water (MS grade), methanol, formic acid (99%), and sodium chloride (≥ 99%) were obtained from VWR, Darmstadt, Germany. Fast Blue B salt (95%) was purchased from MP Biomedicals, Eschwege, Germany. Diammonium hydrogen phosphate (≥ 99%), linolenic acid (99%), *n*-butanol, and diisopropyl ether (≥ 99%) were delivered by Acros Organics, Morris Plains, NJ, USA. Dichloromethane, ethyl acetate, and yeast extract powder (for microbiology) were obtained from Th. Geyer, Renningen, Germany. The culture medium preparation for the bioluminescent *Aliivibrio fischeri* bacteria (DSM-7151, German Collection of Microorganisms and Cell Cultures, Berlin, Germany) is listed elsewhere [24]. The solvent toluene (≥ 99.8%) was provided by Fisher Scientific, Schwerte, Germany. Butyrylcholinesterase (BChE, ≥ 245 U/mg) from equine serum was purchased from SERVA, Heidelberg, Germany, and its substrate 1-naphthyl acetate (≥ 98%) was from AppliChem, Darmstadt, Germany. Nine different convenience tomato products were bought in local markets in Giessen, Germany.

### Preparation of standard mixture, positive control, and simulated intestinal fluid

Stock solutions (10 mg/mL) of the FFAs myristic (C14:0), palmitic (C16:0), stearic (C18:0), oleic (C18:1), linoleic (C18:2), and linolenic (C18:3) acid were prepared in *n*-hexane. The secondary plant metabolites quercetin, naringenin, ascorbic acid, and chlorogenic acid were prepared each as 1 mg/mL stock solution in methanol. A standard mixture was obtained by diluting 100 µL of each FFA, and 1 mL of each secondary plant metabolite stock solution in a 10-mL volumetric flask which was filled with methanol to the mark. The final concentration was 100 µg/mL for each substance. As positive control (PC) solution for the intestinal digestion, rapeseed oil was diluted in *n*-hexane (1 mg/mL). The preparation of the simulated intestinal fluid (SIF) [15], pancreatin solution (panc, 20 mU/µL in SIF) [15] containing bile extract [17], and CaCl_2_ (6 pmol/µL) solution [17] were described elsewhere. The activity of panc was measured as trypsin activity with the *p*-toluene-sulfonyl-l-arginine methyl ester (TAME) assay according to Morlock *et al.* [17].

### Preparation of ten tomato product samples

Nine different convenience tomato products (powdered or liquid) were prepared, and a further tenth (fresh tomato soup) was homemade (Table S1). In case of powdered products, an aliquot was dissolved in the respective volume of water and heated according to each manufacturer instruction to prepare the final product. Liquid products were only heated in a beaker on a heating plate. Each cooled sample (5 g) was mixed with 5 mL *n*-butanol, vortexed for 10 min, and ultrasonicated (Sonores Digiplus, Bandelin, Berlin, Germany) for 15 min. After centrifugation at 3,000 × *g* for 5 min (Labofuge 400, Heraeus, Hanau, Germany), supernatants were filtered through a 0.45 µm cellulose acetate filter (VWR, Darmstadt, Germany) and transferred into autosampler vials.

### NP-HPTLC–nanoGIT^+active^–UV/Vis/FLD–EDA workflow

For the initial studying of HPLC gradients and MS acquisition methods, the standard mixture was applied 40-fold, each as 4 mm × 2 mm-area (500 ng/area), on the HPTLC plate silica gel 60 F_254_ MS grade (Automatic TLC Sampler 4 with Freemode Option of winCATS software version 1.4.7.2018, CAMAG, Muttenz, Switzerland). For the final method, *n*-butanol extracts (5 µL/band) were applied twice as 6-mm bands on pre-washed [8] HPTLC plates silica gel 60 F_254_ MS grade. The PC solution (5 µL/band) was co-applied on the penultimate track. Then, sample tracks and PC were oversprayed according to the on-surface protocol [17] with the bile-extract-containing panc solution (5 µL) and with CaCl_2_ solution (1 µL). Additionally, one track containing only panc and CaCl_2_ was co-applied as negative control (NC). The top 8 cm of the plate were covered with a second plate (layer faced upwards), so that exclusively the application zone was wetted by piezoelectrical spraying of a 0.1 M sodium chloride solution (Derivatizer; 1.25 mL, yellow nozzle, level 6). Both plates [25] were transferred in a moistened polypropylene box (26.5 cm × 16 cm × 10 cm, KIS, ABM, Wolframs-Eschenbach, Germany) with pre-wetted filter-paper lining and incubated for 1 h at 37 °C in an oven (Memmert, Schwabach, Germany). The cover plate remains on the sample plate during incubation to prevent the first 2 cm from running dry through the hygroscopic activity of the dry (but now covered) silica gel. After incubation, the plate was dried for 4 min in a stream of cold air. Plates were developed with *n*-hexane–dichloromethane–methanol–water (40:50:10:1, *V*/*V*/*V*/*V*) up to 70 mm (further studied mobile phases in Table S2 and Fig. S1) in a twin trough chamber (20 cm × 10 cm, CAMAG) and documented under white light (Vis), ultra-violet (UV) 254 nm, and fluorescence light detection (FLD) at 366 nm (TLC Visualizer 3, CAMAG). HPTLC instruments were controlled by visionCATS software (version 3.1.21109.3, CAMAG). Subsequently, effect-directed analysis (EDA) with the *A. fischeri* bioassay as well as α-/β-glucosidase and AChE/BChE enzyme inhibition assays was performed as described [8].

### Heart cut–RP-HPLC–DAD–HESI-HRMS/MS

Bioactive zones were heart-cut eluted from the HPTLC plate using a fully automated autoTLC-MS interface [26, 27] with an oval elution head (4 mm × 2 mm). An HPLC standalone pump (MX010PFT, Teledyne SSI, State College, PA, USA) provided the eluent water–methanol (9:1, *V*/*V*) at a flow rate of 0.1 mL/min. Elution time was set to 60 s, including 20 s backup time. Analytes were transferred through a biocompatible inline filter (IDEX Health & Science, Rohnert Park, CA, USA) to a two-position six-port switching valve (MXT series, PD715-000, Rheodyne, IDEX) with an installed 50-µL sample loop and desalting cartridge (Accucore RP-MS, 10 mm × 2.1 mm, 2.6 μm, Thermo Fisher Scientific) [9]. Analytes were trapped on the cartridge within the first 40 s elution time, until the valve was switched, and the 12-min HPLC gradient transferred the analytes with a flow rate of 0.4 mL/min to the main column (Accucore RP-MS 100 mm × 2.1 mm, 2.6 μm, thermostated at 40 °C, Thermo Fisher Scientific). The gradient consisted of eluent A (water with 2.5 mM ammonium acetate, pH 4.5 adjusted with acetic acid) and eluent B (methanol). Starting conditions were 2% B for 2 min (elution), which were increased to 100% B (2–7 min), and held for 3 min (7–10 min), followed by 2 min equilibration time (gradient optimization in Fig. S2). Note that the autoTLC-MS elution head was automatedly rinsed for 1 min at half-gradient time, when the autoTLC-MS interface was isolated from the pressurized HPLC system. The Dionex Ultimate HPLC system (Dionex Softron, Germering, Germany) was equipped with binary pump (HPG-3200SD), autosampler (WPS-3000TXRS), column oven (TCC-3000RS), and diode array detector (DAD-3000RS), connected to a Q Exactive Plus Hybrid Quadrupole-Orbitrap mass spectrometer, with an Ion-Max HESI-II probe (both Thermo Fisher Scientific). DAD detection was performed at 240 nm, 280 nm, and 320 nm after spectrum recording 200–400 nm. HRMS/MS signals were recorded via the polarity switching full-scan data-dependent MS2 (ddMS2) mode. Ionization settings were equal for all MS acquisition methods: sheath gas 20 AU, aux gas 10 AU, spray voltage 3.5 kV, capillary temperature 320 °C, probe heater temperature 350 °C, S-lens RF level 50 AU. The full-scan settings were mass range of *m*/*z* 100–1100, resolving power of 70,000 (at *m*/*z* 200, full width at half-maximum, FWHM) and automatic gain control (AGC) target 3e6. Fragmentation scans followed in Top5 ddMS2 acquisition mode at a mass range of *m*/*z* 80–1000, resolution of 17,500 FWHM, AGC target 1e6, and stepped normalized collision energy of 20, 40, and 60 eV (further MS acquisition methods in Fig. S3). The mass spectrometer was calibrated weekly with Pierce TM LTQ Velos ESI positive/negative ion calibration solution (Thermo Fisher Scientific). The instrument was controlled and spectra were recorded with Xcalibur 4.2.47 with Foundation 3.1.261.0 and SII for Xcalibur 1.5.0.10747 (Thermo Fisher Scientific).

## Results and discussion

**Fig. 1:**
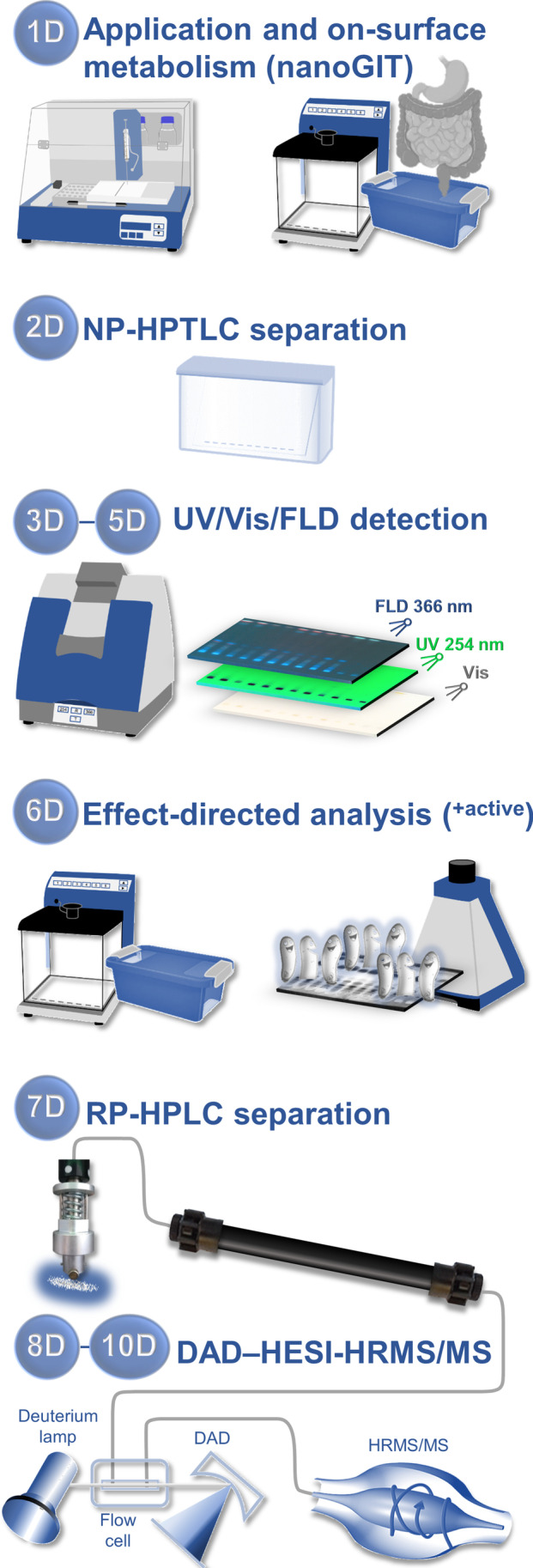
Schematic workflow of the straightforward 10D hyphenated NP-HPTLC−nanoGIT^+active^–UV/Vis/FLD–EDA–heart cut–RP-HPLC–DAD–HESI-HRMS/MS taking 2.5–4 h depending on the assay, or 13–20 min per sample, and consumption costs of approximately 1.50 € per sample, plus 0.15 € per substance elution for heart cut–RP-HPLC–DAD–HESI-HRMS/MS

For the intended development of a proactive qualitative non-target screening strategy, the recently developed nano-molar NP-HPTLC–nanoGIT^+active^ system [17] and eight-dimensional (8D) hyphenation [9] were combined and optimized. Further, the detection was adapted for a high-resolution tandem mass spectrometer. This resulted in a 10D hyphenation, i.e., NP-HPTLC–nanoGIT^+active^–UV/Vis/FLD–EDA–heart cut–RP-HPLC–DAD–HESI-HRMS/MS (Fig. 1). As first dimension (1D), the on-surface digestion of tomato samples with pancreatic enzymes simulated the human gastrointestinal tract metabolism (nanoGIT). Separating side-by-side digested versus non-digested raw extract samples via NP-HPTLC represented the second dimension (2D). Detection at white light illumination (3D), UV 254 nm (4D), and FLD 366 nm (5D) gave information about chromophores, UV-absorbing, and natively fluorescing molecules, respectively. To prioritize compounds among the thousands of unknown compounds in a complex food sample, an effect-directed assay (EDA, ^+active^) was included as sixth dimension (6D). Zones showing bioactivity were heart-cut eluted to an orthogonal RP-HPLC column (7D) and further characterized via DAD (8D) and HRMS/MS (9D/10D). This 10D hyphenation was used to demonstrate the comprehensive analysis of complex samples and metabolomic processes to better understand how bioavailability and bioactivity of compounds are influenced. Also unknown bioactive compounds not in the previous analytical focus can be detected and subjected to simulated static digestion.

Compared to previous studies [8, 9], the technical novelty is the integration of a simulated on-surface digestion step [17] prior to sample separation. Compared to the demonstrated more complex intestinal digestion, the integration of the oral and gastric phases of digestion and phase I and phase II of metabolism is more easily performed. Moreover, the HRMS/MS instrument is superior to the single quadrupole MS system used previously [9]. The high-resolution ability and fragmentation option made the workflow a true non-target analysis, allowing assignment of molecular formula and structural characteristics. The potential for quantification employing an equivalent system setup were investigated elsewhere [28]. The effect-directed detection via an antibacterial bioassay and four enzyme inhibiting assays as well as the method performance, versatility, and robustness were examined using nine convenience tomato products and a freshly prepared homemade tomato soup.

### Mobile phase development for NP-HPTLC–nanoGIT^+active^–UV/Vis/FLD–EDA

As a broad compound spectrum in a wide polarity range was expected in the *n*-butanol extracts of the tomato products, different mobile phases (Table S2 and Fig. S1) were tested to detect a difference between digested versus non-digested raw extract samples. The pancreatic enzyme mixture used for the intestinal digestion contained three classes of enzymes, i.e., lipases, amylases, and peptidases. As classical degradation products, e.g., FFAs from triacylglycerols (TAGs), diacylglycerols, or monoacylglycerols mediated by lipases, mono-, di-, and oligosaccharides from amylolytic cleavage or peptides from proteolytic activity were expected apart from further molecules (glycosides, liposaccharides, lipopeptides, lipoproteins, etc.). Therefore, mobile phase development focused on such cleavage products of those enzymes. Although apolar solvents, such as *n*-hexane, ethyl acetate, and toluene, were considered as basic solvents, others, e.g., dichloromethane, methanol, water, and organic acids, were added to improve selectivity and band sharpness (Table S2). The resulting bioautograms were exemplarily compiled for tomato product sample 8 detected via the *A. fischeri* bioassay (Fig. S1). The *A. fischeri* bioassay indicated the influence of compounds on the bacterial energy metabolism via the detection of bioluminescence reduction but it does not inform on the exact underlying antibacterial mechanism. The mobile phase *n-*hexane-dichloromethane-methanol–water (40:50:10:1, *V*/*V*/*V*/*V*; Table S2, no. 16) was the best choice to separate both the polar and mid polar components at lower *hR*_F_ values as well as the apolar substances in the upper part of the chromatogram. During the separation step, the twin trough chamber had to be closed tightly as the selected mobile phase consisted of highly volatile organic solvents.

### Optimizations for automated non-target RP-HPLC–HESI-HRMS/MS

The latest 8D hyphenation (MS) was extended to a 10D hyphenation (HRMS/MS). Thus, instrumentation had to be changed from an ultra-performance HPLC (waters system) [9] to a standard-pressure HPLC (Dionex system), and adapting the column gradient to an instrument that can sustain less pressure was challenging. The used Dionex system has an upper pressure limit of only 62 MPa (620 bar), and in addition, the backpressure from the HESI probe was higher than for the previously employed Waters ESI probe [9]. To prevent the instrumentation from working on its limit, the gradient was adjusted to the given circumstances (Fig. S2). The applied standard mixture was heart-cut eluted with a previously optimized water–methanol mixture (9:1, *V*/*V*) [9] to RP-HPLC–DAD–HESI-HRMS/MS. The eluents (A: 2.5 mM ammonium acetate adjusted to pH 4.5 with acetic acid; B: methanol) remained the same. The gradient program of the previous 8D-hyphenation [9] was first tested at half of the flow rate. As a result, the peak shapes were not satisfactory, adjacent peaks were not baseline-separated, and FFAs did not elute at all from the RP column in the given time (Fig. S2a). Thus, the organic gradient portion was raised to 100%, and the duration was shortened to 10 min; however, baseline separation and the elution of the most apolar stearic acid (C18:0) were not satisfactory (Fig. S2b). A less steep increase in organic solvent (Fig. S2c), a longer time holding at 100% B, and a higher flow rate of 0.4 mL/min (Fig. S2d) finally completed the gradient optimization.

**Fig. 2:**
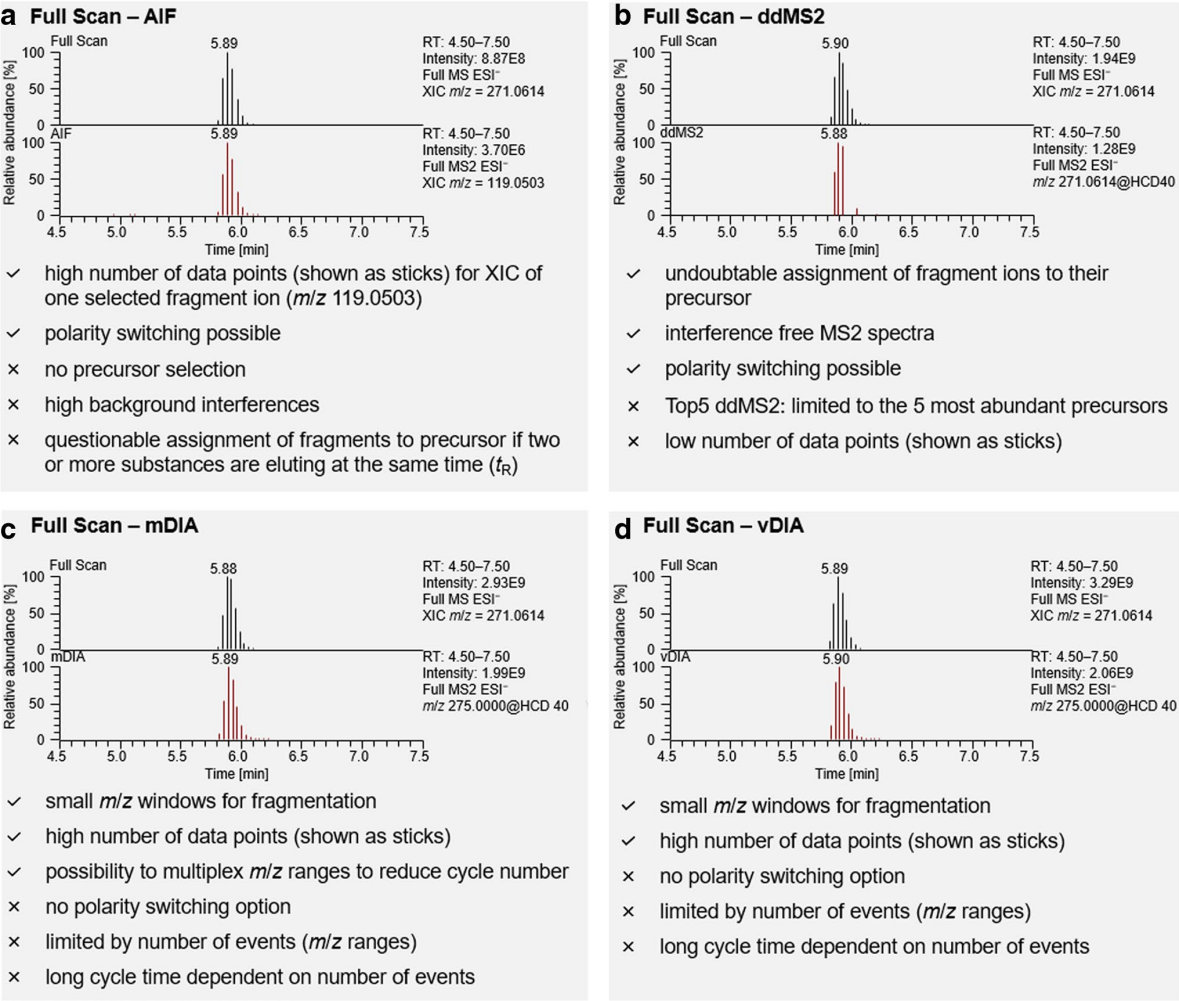
Study of HRMS acquisition modes: extracted ion chromatograms (XIC) from full scan and respective MS2 chromatograms from different acquisition modes analyzed by a Q Exactive Plus Hybrid Quadrupole-Orbitrap. Naringin (500 ng/area each) applied on an HPTLC silica gel 60 F_254_ MS grade plate was eluted with water–methanol (9:1, *V*/*V*) to RP-HPLC–HESI-HRMS/MS. The full scans (displayed as stick) of the deprotonated naringin (*m*/*z* 271.0614, [M − H]^−^) were combined with **a** all ion fragmentation (AIF, product ion spectra of *m*/*z* 199.0503 C_8_H_7_O^−^), **b** data-dependent MS2 (ddMS2, directly from the isolated precursor), **c** multiplexed data independent acquisition (mDIA), and **d** variable DIA (vDIA) both latter from the MS2 scan event *m*/*z* 250–300

To evaluate the best non-target acquisition settings for hybrid Quadrupole-Orbitrap, all ion fragmentation (AIF) and ddMS2 fragmentation were employed in the polarity switching mode and compared with multiplexed and variable data-independent acquisitions (m/vDIA) in the single polarity mode. The detailed parameter settings in the different acquisition modes are summarized (Fig. S3). Full-scan extracted ion chromatograms (XIC) and corresponding MS2 scan events at higher-energy collisional dissociation (HCD) are exemplarily shown for the flavonoid naringenin at *m*/*z* 271.0614 [M − H]^−^ (Fig. 2). A drawback of AIF acquisition (Fig. 2a) is that no precursor molecule is selected for fragmentation and all ions were transferred to the collision cell at the same time resulting in high background ion interference [3]. Furthermore, the assignment of fragments to a precursor cannot be made unambiguously for several analytes eluting at the same time. The shown XIC MS2 chromatogram at *m*/*z* 119.0503 (C_8_H_7_O^−^) is based on a known fragment of naringenin, which also illustrates the lack of potential as a non-target screening acquisition method.

In contrast, data-dependent analysis (Fig. 2b) obliterated all disadvantages mentioned for AIF. For non-target data acquisition, no inclusion list is available to determine the precursors for fragmentation [3]. Hence, the used Top5 ddMS2 method isolated the five most abundant precursors in each full-scan event and transferred them to the collision cell. These settings entailed advantages and disadvantages at the same time. Fragments could be assigned to a precursor, and fragmentation spectra are almost interference-free. Still, the method is limited to only five precursors which could be fragmented at the same time, neglecting low abundant signals. This is a limitation since active compounds that are hardly ionizable but responsible for the detected bioactivity in the transferred zone can thus be overlooked. Further, the low MS2 scan rate (only four fragmentation scans, Fig. 2b) was additionally minimized by polarity switching, which is, however, indispensable for non-target analysis.

In contrast, DIA methods (Fig. 2c, d) are suitable for non-target data acquisition, but cycle time is the limiting factor disabling polarity switching [5, 29]. The missing opportunity for polarity switching in both DIA acquisition methods is therefore their main drawback. Compared to AIF, smaller *m*/*z* ranges were isolated by the quadrupole (Fig. S3, isolation window) and guided to the fragmentation cell. A complete scan cycle consisted therefore of a full scan followed by the DIA *m*/*z* windows which were worked off in sequence [3, 4, 29]. The smaller the isolation windows, the longer the cycle time. In mDIA acquisition mode, the isolation window is fixed for a certain mass range (Fig. S3, MSX ID 9 with *m*/*z* 500.00000–550.00000 and 550.00000–600.00000), but several *m*/*z* ranges could be simultaneously fragmented and analyzed by multiplexing. Since the bioactive compounds in the tomato products were expected to be small molecules of low molecular weight, the higher *m*/*z* ranges were multiplexed, and the lower *m*/*z* ranges were left singly to provide interference-free MS2 spectra. In vDIA acquisition mode, multiple isolation windows were selected, in which the isolation windows for higher *m*/*z* values were set wider than for lower *m*/*z* for the same reasons mentioned above.

Since DIA lacks polarity switching and acquisition is always a decision between resolution and scan speed as well as width of isolation windows and number of MS2 scan events [29], it was discarded as a non-target screening acquisition method. Since AIF acquisition is too impurity-prone and non-selective, it was also discarded as an option. Hence, a ddMS2 acquisition method was selected for automated non-target RP-HPLC–HESI-HRMS/MS analysis of the eluted zones.

### Non-target food screening via NP-HPTLC–nanoGIT^+active^–UV/Vis/FLD–EDA–heart cut–RP-HPLC–DAD–HESI-HRMS/MS

**Fig. 3:**
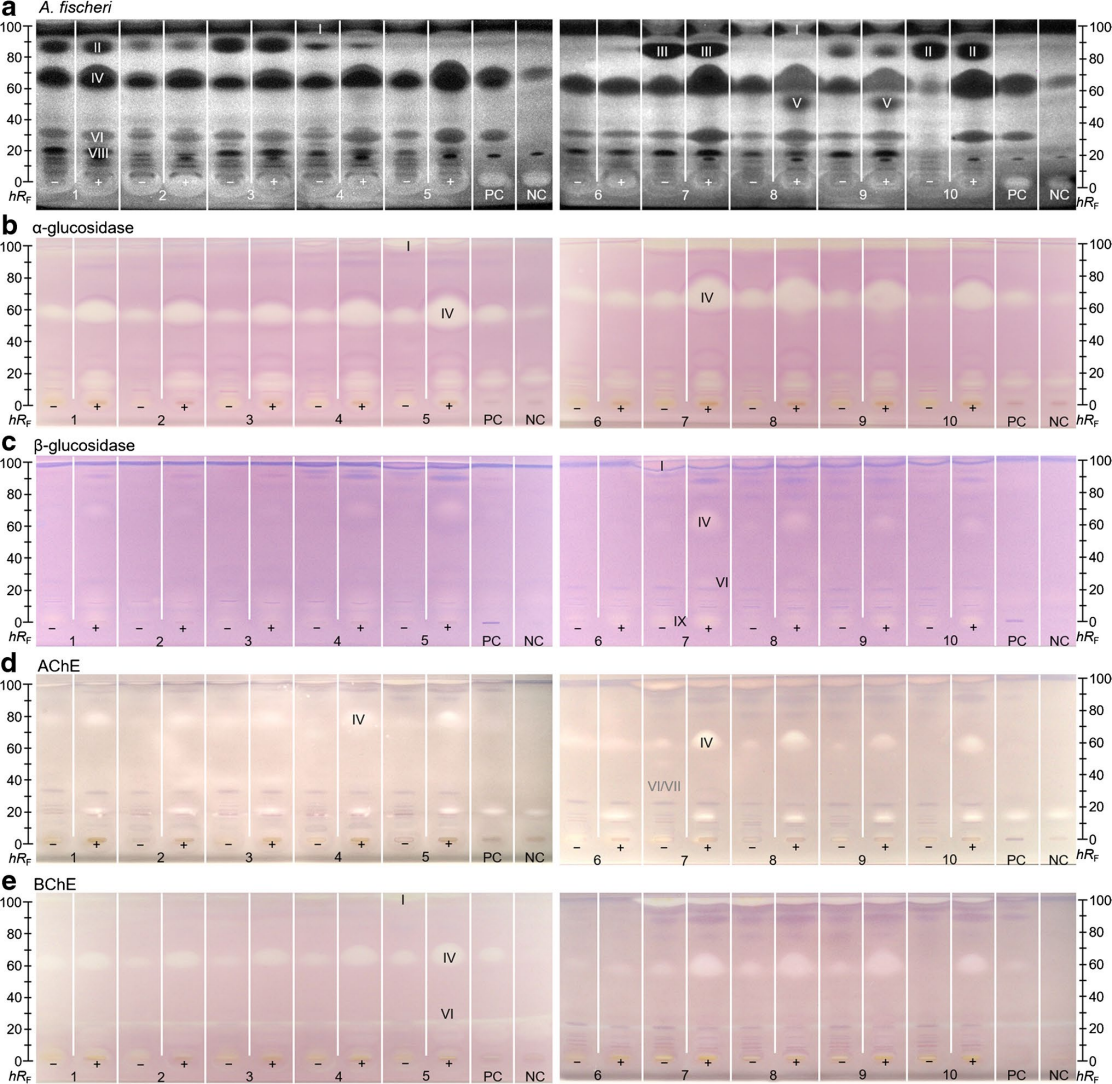
Food screening for bioactive compounds: NP-HPTLC−nanoGIT^+active^–UV/Vis/FLD–EDA profiles of non-digested (−) *versus* panc-digested (+) raw extracts of tomato products (5 µL/band each, assignments in Table S1) along with positive control (PC, digested rapeseed oil) and negative control (NC, pancreatic enzyme mix and bile salts) on HPTLC plate silica gel 60 F_254_ MS grade, developed with *n*-hexane–dichloromethane–methanol–water (40:50:10:1, *V*/*V*/*V*/*V*) up to 70 mm and detected via the bioluminescence (depicted as a greyscale image) after the *A. fischeri* bioassay (**a**), and at white light illumination after the α-glucosidase (**b**), β-glucosidase (**c**), acetylcholinesterase (**d**), and butyrylcholinesterase (**e**) inhibition assays. Bioactive zones I − IX were heart-cut eluted for further characterization by RP-HPLC–DAD–HESI-HRMS/MS. *hR*_F_ values were dependent on plate activity, as evident for zone IV (**d**)

**Fig. 4:**
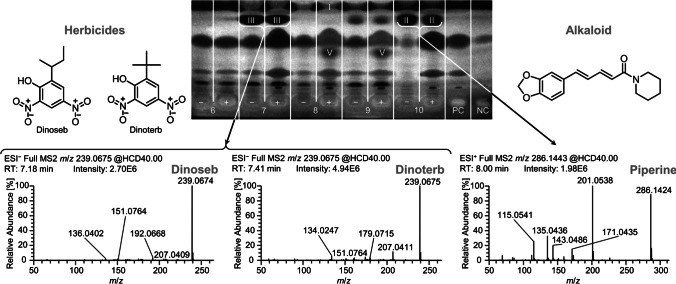
Recording of NP-HPTLC−nanoGIT^+active^–UV/Vis/FLD–EDA–heart cut–RP-HPLC–DAD–HESI-HRMS/MS spectra of zones II and III, both at *hR*_F_ 81 ± 1

One aliquot of each tomato product extract was used directly (non-digested), whereas the other aliquot was metabolized by simulated static pancreatic digestion (1D). After their separation (2D), the UV/Vis/FLD chromatograms (3D–5D) showed only a few zones (Fig. S4). For the bioactivity detection (6D), the *A. fischeri* bioassay (Fig. 3a) was selected due to its simplicity and more universal detectability. Further, glucosidase (Fig. 3b, c) and cholinesterase (Fig. 3d, e) inhibition assays were employed to focus on the relationship between the diet and mainstream civilization diseases, e.g., diabetes type II and Alzheimer’s [8]. After bioactivity screening, the most prominent zones I–IX (Figs. 3 and 4) were heart-cut eluted and analyzed via RP-HPLC–DAD–HESI-HRMS/MS (7D–10D). For tentative assignment of the molecules, database and literature research was ran using open source databases, i.e.*,* PubChem (https://pubchem.ncbi.nlm.nih.gov), ChemSpider (www.chemspider.com), MetFrag (https://msbi.ipb-halle.de/MetFragBeta), FOODB (https://foodb.ca), and NIST WebBook (https://webbook.nist.gov).

Comparing the effect-directed profiles of non-digested (−) and digested (+) raw extracts of convenience tomato product samples 1–9 (Fig. 3) only slight differences were detectable due to the same main ingredient (tomatoes). Zone I (*hR*_F_ 99 ± 1) exhibited bioactivity through all assays but did not show any significant HRMS signals. Presumably, lipids, e.g., TAGs, from vegetable oils were chromatographed to the solvent front. Missing lipid signals in HRMS/MS were explained by their high apolar properties, which prohibited them to elute from the plate with 90% water. It was assumed that zone I is partially metabolized by the pancreatic enzymes. Sample 5 (Fig. 3b) illustrated this hypothesis. While zone I (*hR*_F_ 99 ± 1) is very intensive in the non-digested raw extract 5 (−), the α-glucosidase inhibition was weaker after intestinal digestion in the digested sample 5 (+). In contrast, zone IV (Fig. 3, *hR*_F_ 55–80, depending on plate activity) significantly increased through digestion. This intensification was detected through all assays, suggesting products of metabolism. The recording of HRMS signals revealed a whole pattern of FFAs with chain lengths from C12–C18 (Table 1) as possible degradation products of the TAGs cleavage via the pancreatic lipases. Myristic, palmitic, stearic, oleic, linoleic, and linolenic acid were not separated by NP-HPTLC, but by the orthogonal RP-HPLC. A remarkable difference comparing convenience products with the self-made tomato soup is the comparatively very low content of FFAs in the non-digested raw extract of the self-made tomato soup. The freshly prepared food contained fewer TAG degradation products which indicated a good quality of the oil used [39].

**Table 1:** Tentative signal assignments of bioactive zones I−IX in tomato products without (−) and with (+) simulated intestinal on-surface digestion obtained by NP-HPTLC−nanoGIT^+active^–UV/Vis/FLD–EDA–heart cut–RP-HPLC–DAD–HESI-HRMS/MS (n.d., not detected; bold, most abundant fragment ion; sh, shoulder)

Zone	Sample	Observed effect	*hR*_F_ (± 1)	*t*_R_ [min]	Molecular formula	Ion species	Precursor [*m*/*z*]	Δ*m*/*z* [ppm]	MS2 fragments [*m*/*z*]	λ_max_ [nm]	Tentative assignment	Lit.
I	1–5(− / +) 7–10(− / +)	*A. fischeri* active (enhancing), of AChE/BChE and α-/β-glucosidase inhibition	99	n.d.								
II	1(− / +) 10(− / +)	*A. fischeri* active	82	7.77	C_17_H_19_O_3_N	[M + H]^+^ [M + Na]^+^ [2 M + H]^+^ [2 M + Na]^+^ [2 M + K]^+^	286.1438 308.1254 571.2805 593.2622 609.2362	0.00 0.92 − 0.38 0.08 − 0.07	**201.0540**^+^ 143.0488^+^ 135.0437^+^ 115.0541^+^	245, 330	Piperine	[30]
III	7(− / +)	*A. fischeri* active	80	7.21	C_10_H_12_O_5_N_2_	[M − H]^−^	**239.0675**	− 0.64	207.0409^−^ 192.0668^−^ 136.0402^−^	−	Dinoseb (pesticide)	[31]
				7.43	C_10_H_12_O_5_N_2_	[M − H]^−^	**239.0675**	− 0.64	207.0411^−^ 179.0714^−^ 151.0764^−^ 134.0247^−^	−	Dinoterb (pesticide)	[31]
IV	all(− / +) PC	*A. fischeri* active, AChE/BChE and α-/β-glucosidase inhibition	55–80	9.00	C_14_H_28_O_2_	[M − H]^−^	227.2018	− 0.55	no fragments	−	Myristic acid	[32]
				9.00	C_18_H_30_O_2_	[M − H]^−^	277.2174	− 0.38	no fragments	−	Linolenic acid	[32]
				9.22	C_18_H_32_O_2_	[M − H]^−^	279.2332	− 0.99	no fragments	−	Linoleic acid	[32]
				9.33	C_16_H_32_O_2_	[M − H]^−^	255.2331	− 0.53	no fragments	−	Palmitic acid	[32]
				9.33sh	C_18_H_34_O_2_	[M − H]^−^	281.2488	− 0.73	no fragments	−	Oleic acid	[32]
				9.72	C_18_H_36_O_2_	[M − H]^−^	283.2646	− 1.04	no fragments	−	Stearic acid	[32]
V	8(+) 9(+)	*A. fischeri* active	49	8.31	C_10_H_20_O_2_	[M − H]^−^	171.1391	− 0.27	no fragments	−	Capric acid	[33]
				8.67	C_12_H_24_O_2_	[M − H]^−^	199.1704	− 0.43	no fragments	−	Lauric acid	[33]
VI	all(− / +) PC	*A. fischeri* active, AChE/BChE and α-/β-glucosidase inhibition	28	7.38	C_15_H_26_O_5_	[M − H]^−^ [M + Cl]^−^ [M + HCOO]^−^ [M + H_3_C–COO]^−^ [M − H_2_O + H]^+^ [M + H]^+^ [M + NH_4_]^+^ [M + Na]^+^ [M + K]^+^	285.1710 321.1476 331.1765 345.1920 269.1746 287.1852 304.2118 309.1669 325.1409	− 0.74 − 0.48 − 0.85 − 0.32 0.47 0.53 0.13 1.05 0.74	**211.1338**^−^ 183.1388^−^ **269.1744**^+^ 195.1379^+^ 149.1325^+^	−	Unknown (diglycerides/oxidated monoglycerides)	
VII	7(−)	weak inhibition of AChE	28	6.11	C_16_H_31_O_7_N	[M − H]^−^ [M + Cl]^−^ [M + HCOO]^−^ [M + H_3_C–COO]^−^ [M + H]^+^ [M + Na]^+^ [M + K]^+^	348.2032 384.1801 394.2087 408.2243 **350.2174** 372.1994 388.1733	− 1.07 − 1.70 − 1.10 − 1.03 − 0.31 − 0.44 − 0.33	**274.1663**^−^ 226.1451^−^ 208.1344^−^ 91.0401^−^ 104.0711^+^	−	Unknown	
				6.63	C_12_H_22_O_5_	[M − H]^−^ [M + Cl]^−^ [M + HCOO]^−^ [M + H_3_C–COO]^−^ [M − H_2_O + H]^+^ [M + NH_4_]^+^ [M + Na]^+^ [M + K]^+^	245.1395 281.1163 291.1453 305.1608 229.1431 264.1801 269.1353 285.1093	− 0.09 − 0.65 − 1.14 − 0.76 1.55 1.74 2.44 1.90	**171.1026**^−^ 173.1164^+^ 155.1059^+^ **109.1009**^+^ 67.0547^+^	−	Unknown (diglycerides/oxidated monoglycerides)	
				6.63	C_13_H_26_O_6_	[M + Cl]^−^ [M + HCOO]^−^ [M + H_3_C–COO]^−^ [M + NH_4_]^+^ [M + Na]^+^ [M + K]^+^	313.1426 323.1713 337.1870 296.2062 301.1617 317.1355	− 0.73 − 0.33 − 0.64 1.84 1.66 1.88	**269.1362**^+^ (sodium fragment)	−	Unknown (diglycerides/oxidated monoglycerides)	
				7.08	C_14_H_24_O_5_	[M − H]^−^ [M + Cl]^−^ [M + HCOO]^−^ [M + H_3_C–COO]^−^ [M − H_2_O + H]^+^ [M + H]^+^ [M + NH_4_]^+^ [M + Na]^+^ [M + K]^+^	271.1554 307.1321 317.1607 331.1766 255.1590 273.1695 290.1962 295.1515 311.1254	− 1.18 − 0.99 − 0.44 − 1.24 0.30 0.48 0.03 0.26 0.39	233.8611^−^ **197.1183**^−^ 169.1232^−^ 181.1223^+^ 135.1170^+^ 107.0859^+^ 93.0704^+^	−	Unknown (diglycerides/oxidated monoglycerides)	
VIII	1–9(− / +)	*A. fischeri* active	18	6.99	C_15_H_12_O_5_	[M − H]^−^ [M + Cl]^−^	**271.0614** 307.0381	− 0.89 − 0.79	177.0192^−^ 151.0036^−^ 119.0501^−^ 107.0138^−^	215, 290	Naringenin	[34]
IX	7(−)	Inhibition of β-glucosidase	0	5.13	C_14_H_25_O_3_N_3_	[M − H]^−^ [M + Cl]^−^ [M + H]^+^ [M + Na]^+^ [M + K]^+^	282.1825 318.1591 **284.1972** 306.1791 322.1530	− 0.62 − 0.33 − 1.06 − 0.81 − 0.69	159.1026^−^ **140.0829**^−^ 123.0564^−^ 105.0457^−^ 266.1867^+^ 124.0873^+^ 107.0608^+^ 81.0455^+^	−	Unknown	
				5.13	C_21_H_32_O_10_	[M − H]^−^ [M + Cl]^−^ [M + H]^+^ [M + NH_4_]^+^ [M + Na]^+^ [M + K]^+^	**443.1926** 479.1693 445.2076 462.2341 467.1898 483.1634	− 0.83 − 0.75 − 1.76 − 1.61 − 2.14 − 1.33	265.1435^+^ 247.1324^+^ (ammonia fragments)	−	Unknown	
				5.30	C_18_H_28_O_9_	[M − H]^−^ [M + Cl]^−^ [M + NH_4_]^+^ [M + Na]^+^ [M + K]^+^	**387.1666** 423.1428 406.2073 411.1626 427.1368	− 1.42 − 0.13 − 0.34 − 0.03 − 0.74	163.1128^−^ 59.0133^−^ **209.1175**^+^ 191.1068^+^ 149.0963^+^ 131.0858^+^	220, 280	Tuberonic acid glucoside	[35]
				5.87	C_19_H_30_O_8_	[M + Cl]^−^ [M + HCOO]^−^ [M + H_3_C–COO]^−^ [M + H]^+^ [M + NH_4_]^+^ [M + Na]^+^ [M + K]^+^	421.1640 431.1925 445.2082 387.2015 404.2282 409.1836 425.1576	− 1.16 − 0.51 − 0.69 − 0.37 − 0.85 − 0.68 − 0.88	No fragments	−	Unknown	
				6.24	C_27_H_30_O_16_	[M − H]^−^ [M + Cl]^−^ [M + H]^+^ [M + Na]^+^ [M + K]^+^	609.1469 645.1235 611.1613 633.1433 649.1173	− 1.21 − 1.04 − 1.05 − 1.12 − 1.21	**301.0713**^−^ **303.0501**^+^	260, 355	Rutin	[36]
*-*	NC	*A. fischeri* active, α-glucosidase inhibition	65	9.36	C_16_H_32_O_2_	[M − H]^−^	255.2330	− 0.14	No fragments	−	Palmitic acid	
				9.72	C_18_H_34_O_2_	[M − H]^−^	283.2642	0.26	No fragments	−	Stearic acid	
*-*	NC	*A. fischeri* active, AChE and α-glucosidase inhibition	15	8.05	C_24_H_38_O_4_	[M − H]^−^ [M + Cl]^−^ [M + HCOO]^−^ [M + H3C-COO]^−^ [2 M − H]^−^ [M − 2H2O + H]^+^ [M − H2O + H]^+^ [M + H]^+^ [M + NH4]^+^ [M + K]^+^ [2 M + NH4]^+^	389.2703 425.2471 435.2757 449.2914 779.5477 355.2636 373.2742 391.2847 408.3113 429.2406 798.5891	− 1.33 − 1.59 − 1.12 − 1.22 − 1.26 − 1.31 − 1.23 − 1.16 − 1.09 − 0.89 − 1.51	No fragments	−	Component of bile acids (7-ketolithocholic acid)	[37]
				8.46	C_24_H_40_O_4_	[M − H]^−^ [M + Cl]^−^ [M + HCOO]^−^ [M + H3C-COO]^−^ [2 M − H]^−^	391.2856 427.2621 437.2910 451.3067 783.5783	− 0.48 − 0.09 − 0.31 − 0.39 − 0.35	No fragments	−	Components of bile acids (hydeoxycholic acid, ursodeoxycholic acid, chenodeoxycholic acid, deoxycholic acid)	[37, 38]

The importance of the second orthogonal chromatography was once more highlighted by the following example. At *hR*_F_ 81 ± 1 in samples 1–4, 7, 9, and 10, in both digested and non-digested samples, the two antibacterial zones II and III were observed in the *A. fischeri* bioautogram (Fig. 3a). The bioactive zone at *hR*_F_ 81 ± 1 of samples 1–4, all containing pepper or seasoning, was caused by piperine and assigned as zone II. In the self-made tomato soup (sample 10), the antibacterial response of zone II is unequivocally affiliated with piperine (Table 1), the main alkaloid in black pepper [30]. Regardless of a slightly different zone shape but being at a similar migration distance, zone III in sample 7 could be assumed to be also piperine, but the ingredients list (Table S1) neither showed pepper, spices, nor any declaration indicating the alkaloid. Instead, the two herbicides dinoseb and dinoterb were found (Table 1, Fig. 4). According to European Commission Regulation EU 2015/868 [31], maximum residue levels for the herbicides are 0.02 mg/kg each in fruiting vegetables (tomatoes), berries and small fruits (wine grapes), and bulb vegetables (onion and garlic). All these ingredients were present in sample 7. Since the *A. fischeri* bioassay and the HRMS/MS analysis are very sensitive, even traces of dinoseb and dinoterb can be found in the sample because authorized herbicides are normally highly active and potent. Neither both herbicides nor the piperine alkaloid were influenced by the on-surface intestinal digestion.

In the *A. fischeri* bioautogram (Fig. 3a), an extra zone V (*hR*_F_ 49 ± 1) was visible after digestion, exclusively for samples 8 and 9. Short-chain FFAs, such as capric (C10:0) and lauric (C12:0) acid were found in this zone (Fig. 3a, Table 1) formed by enzymatic digestion of TAGs of animal origin [33]. Chromatographic retention behavior and bioactivity against *A. fischeri* of FFAs (C10:0–C18:3), monoacylglycerol, diacylglycerol, and TAG standards were previously confirmed in the same chromatographic system [37]. Comparing the ingredients lists (Table S1), samples 5, 8, and 9 contained cream and skim milk powder as sources of animal fat, which explained the findings. In sample 5, the skim milk powder was added in a lower quantity explained by the higher tomato content (89% tomatoes versus only 48% and 56% in samples 8 and 9, respectively).

Zones VI and VII (both *hR*_F_ 28 ± 1, Fig. 3a, c, d) revealed several signals, but could not be assigned to any ingredient listed. Calculated molecular formulas C_15_H_26_O_5_, C_12_H_22_O_5_, C_14_H_25_O_5_, and C_13_H_26_O_6_ (Table 1) indicated partially oxidized mono- or diacylglycerols. This was plausible as mono- and diacylglycerols are used as emulsifying agents in convenience tomato products. This assumption was also supported by the presence of these zones in the digested rapeseed oil (PC, *hR*_F_ 28) as well as their increase in the digested samples and their absence in the self-made freshly prepared tomato product. Recently, especially the monoacylglycerols were proven to act antibacterial against *A. fischeri* [37].

The flavonoid naringenin caused the antibacterial effect of zone VIII (*hR*_F_ 18 ± 1) in the *A. fischeri* bioautogram (Fig. 3a, Table 1). The bacterial inhibition response was pronounced for samples 1, 3, 4, 6–9, weak for samples 2 and 5, and absent for sample 10. The flavonoid content in tomatoes is dependent on the stage of maturation. Ripe tomato fruits have a high amount of flavonoids accumulated in the peel [34, 36]. Since the tomatoes were peeled for the self-made soup, it was expected that no naringenin was found in sample 10.

The β-glucosidase inhibiting zone IX (*hR*_F_ 0, Fig. 3c, Table 1) was assigned to tuberonic acid glucoside [35] and rutin [36]. Due to the amylolytic activity of pancreatin, tuberonic acid glucoside is expected to be metabolized to its aglycon tuberonic acid. Since rutin is a quercetin-3-rutinoside and pancreatic amylases are not able to cleave rutinosides, the appearance of this flavonoid is also estimated in the metabolized (+) samples.

Signals visible in the NC showing antibacterial, AChE, and α-glucosidase activity were analogously analyzed via heart cut–RP-HPLC–ESI-HRMS/MS. Results showed the expected ingredients (Table 1). Several bile acids (7-ketloithocholic acid, hyodeoxycholic acid, ursodeoxycholic acid, chenodeoxycholic acid, and deoxycholic acid, Table 1) from the porcine bile extract were detected at *hR*_F_ 15 [37, 38]. Further, small amounts of FFAs, more precisely palmitic and stearic acid (Table 1) were also detected in the crude procine bile extract.

More phytochemicals expected in tomatoes, e.g., caffeic acid and coumaric hexosides [40] or other secondary metabolites [41], were presumably less extracted by the apolar extraction solvent *n*-butanol. Adapting the extraction protocol to more polar solvents or consecutive extraction with solvents of different polarities or applying directly the diluted tomato products could enlarge the analyte range. However, these options were not investigated in this study.

### Discussion of the prioritized bioactive compounds

The 10D hyphenation strategy successfully provided an efficient non-target food screening, exemplarily shown for tomato products. Due to the detection of biological effects and prioritization of important bioactive compounds, it was straightforward and more proactive regarding food safety and consumer protection compared to the analytical status quo. As the sample amount applied was the same for all assays, the bioactivity responses can directly be compared on the images. All studied tomato samples showed a similar bioprofile due to the same main ingredient (tomatoes) and possessed health-promoting constituents with the potential to prevent or at least curtail civilization diseases such as Alzheimer’s or diabetes type II. Most striking were the antibacterial, then α-glucosidase, and then BChE inhibition activities among the five different activity mechanisms studied. The effects were observed for an extracted 5-g sample portion taken out of a 250-g meal and considered to be meaningful since the 50-fold effect response has to be imagined to be taken up with a meal. In terms of bioactivity and health benefits, the quality of the convenience tomato products 1–9 can compete, especially after intestinal digestion, with the self-made tomato soup sample 10, which is commonly supposed to be the healthier alternative.

The most pronounced and most versatile effects were observed for the FFAs (zone IV). This effect substantially increased after the simulated intestinal digestion. The self-made tomato soup sample 10 showed ab initio the lowest content of FFAs since it was freshly prepared, but after the simulated intestinal digestion, it was similar in the effect responses to the convenience products. FFAs are known to influence health and disease status of humans. Particularly, the antibacterial effect against both Gram-negative and Gram-positive bacteria was emphasized [42–44]. Despite various ways of acting against pathogens [42, 44], FFAs support to balance the gut microbiota [45]. The cholinesterase inhibition capacities of myristic, oleic, palmitic, and stearic acid were already proved against standards [46]. In context with Alzheimer’s disease, the inhibition of acetylcholine breakdown via AChE and BChE is one therapeutic option to slow down symptoms [47].

Piperine (zone II) and the two co-eluting dinitrophenol herbicides dinoseb and dinoterb (zone III) were also prominent in their antibacterial activity against the *A. fischeri* bacteria. Only piperine has been reported so far to act against human pathogens [48]. The piperine, almost 100% absorbed, does not undergo metabolic alterations [49], which is consistent with our results. Also the herbicides were not influenced by the on-surface intestinal digestion and thus can stay active inside our body. The cellular uptake of herbicides is dependent on the gut microbiota composition, whereby the intestinal absorption of pesticides decreased in presence of microbiota [50].

Further, the health-promoting effects of naringenin (zone VIII) were reported including antidiabetic effects [51]. The antimicrobial properties of the flavonoid were studied against several model organisms [52] but not explicitly *A. fischeri*. The partially oxidized mono- or diacylglycerols (zones VI/VII), but also the FFAs (zone IV), should be tested for the presence of epoxidized forms in future since healthy oils rich in unsaturated fatty acids were recently found to be genotoxic [53].

Considering the enormous bioactive potential of our food, it is known that substances can show more than one effect. The chlorogenic acid and its derivatives is known to have antioxidant [54], anti-inflammatory [8, 55], and anti-HIV [56] properties and also showed anti-diabetes, anti-Alzheimer’s, and anti-tyrosinase activity in our recent publication [8]. To verify the screening result of a multi-potent molecule, the purchase of the detected molecule of interest and co-chromatography including the detection via the indicated assays need to be performed. Further, the exclusion of false-positive signals (physico-chemical interference) or pan-assay interference compounds (PAINS) can easily be clarified on surface via multiplex assays [57, 58].

The used pancreatin enzyme mixture mainly contained lipases, proteases, and amylases [59]. Although the more complex intestinal phase digestion was simulated, the oral and gastric phase as well as the microbiota and phase I and II metabolism need also to be studied to get the full image of the whole digestive processes and metabolism. It has to be taken into account that part of the phytochemicals are chemically modified and excreted, e.g., glucurinated [60] or sulfated [61].

Since crude extracts were analyzed, the separation capacity of HPTLC is not sufficient to separate the samples with adequate resolution, although the detection used for prioritization of compounds can be very specific. The more important was the second orthogonal chromatographic dimension to separate any co-eluting compounds of the first chromatographic dimension.

## Conclusion

The 10D hyphenation showed to be a robust tool for the screening of tomato products for bioactive compounds and their intestinal conversion. Its potential has been impressively demonstrated to better understand complex metabolomic processes regarding bioavailability and bioactivity. The overall workflow allowed parallel screening of six digested and six undigested samples in a single chromatographic run (one plate development). Thus, the overall workflow was cost-saving (approximately 1.50 € consumption costs per sample, plus 0.15 € per substance elution for heart cut–RP-HPLC–DAD–HESI-HRMS/MS) and time efficient (2.5–4 h depending on the assay, or 13–20 min per sample). Costs of method development/optimization/validation or equipment maintenance or CO_2_ footprint were not integrated. The hyphenation of on-surface digestion, bioactivity screening, two-dimensional (heart-cut) orthogonal chromatographic separation, and compound identification, provided substance prioritization and comprehensive information on bioactive components in food and how they might alter through metabolism in the human body. Especially the determination of traces of the two dinitrophenol herbicides dinoseb and dinoterb (zone III) underlines the enormous non-target and proactive potential of the presented 10D hyphenation for food safety. Recent studies on meal replacement products showed that the highly streamlined hyphenation strategy is also transferable to other food matrices and applicable for a wide range of samples. Future studies could include extraction workflows adapted to eating habits, the influence of the microbiota, and the quantification of prioritized compounds to ensure compliance with specific maximum residue levels in foodstuffs.

### Supplementary Information

Below is the link to the electronic supplementary material.

Supplementary file1 (PDF 1339 KB)
