# Supplementary material for: Towards non-target proactive food safety: identification of active compounds in convenience tomato products by ten-dimensional hyphenation with integrated simulated gastrointestinal digestion
Source: Anal Bioanal Chem. 2023 Mar 29;416(3):715–31. doi: 10.1007/s00216-023-04656-0 (PMC10766732; doi:10.1007/s00216-023-04656-0)
Supplement: Supplementary file 1 — Supplementary file1 (PDF 1339 KB) [file 216_2023_4656_MOESM1_ESM.pdf]

Supplementary Information

Towards non-target proactive food safety: Identification of active compounds in convenience tomato products by ten-dimensional hyphenation with integrated simulated gastrointestinal digestion

Tamara Schreiner, Naila M. Eggerstorfer, Gertrud E. Morlock*

Justus Liebig University Giessen, Institute of Nutritional Science, Chair of Food Science, Heinrich-Buff-Ring 26-32, 35392 Giessen, Germany

*Corresponding author. Tel.: +49 641 9939141, fax: +49 641 9939149, E-mail address: Gertrud.Morlock@uni-giessen.de (G.E. Morlock)

Table of contents

Page S–4	**Table S1.** List of the ten investigated tomato products extracted with 5 mL *n*-butanol and filtered through 0.45 µm cellulose acetate filter.
Page S–7	**Table S2.** Investigation of mobile phase systems for tomato extract separation on HPTLC silica gel 60 F_{254} MS-grade plates up to a migration distance of 75 mm (*70 mm).
Page S–8	**Fig. S1.** Investigation of mobile phase systems (no. 1–24, Table S2) on HPTLC plates silica gel 60 F_{254} MS-grade for the NP-HPTLC−nanoGIT$^{+active}$–*Aliivibrio fischeri* separation of the raw (−) *versus* panc-digested (+) extract of tomato product no. 8 (Table S1) along with positive control (PC, rapeseed oil) and negative control (NC, enzyme mix and bile salts) applied (all 5 µL/band each), developed and detected via the bioluminescence (depicted as greyscale image) after the *A. fischeri* bioassay.
Page S–9	**Fig. S2.** Development of the RP-HPLC gradient consisting of eluent A (2.5 mM ammonium acetate pH adusted with acetic acid to 4.5) and eluent B (methanol) using standard mixture (500 ng/area, 4 mm × 2 mm) applied on HPTLC silica gel 60 F_{254} MS-grade plate and heart-cut eluted with water – methanol (9:1 *V*/*V*) to the column and HRMS showing the total ion current (TIC) chromatogram in the negative ionization mode.
Page S–10	**Fig. S3.** RP-HPLC–HESI-HRMS/MS settings for the evaluation of a non-target acquisition strategy. Full scan settings were maintained while MS2 settings were varied as all ion fragmentation (AIF), data-dependent MS2 (ddMS2), multiplexed data-independent acqusition (mDIA), and variable data-independet acquisition (vDIA).

Page S–11	**Fig. S4.** NP-HPTLC−nanoGIT$^{+active}$–UV/Vis/FLD profiles of 10 raw (−) *versus* panc-digested (+) tomato products (5 µL/band, Table S1) along with positive control (PC, rapeseed oil), and negative control (NC, pancreatic enzyme mix and bile salts) on HPTLC silica gel 60 F_{254} MS-grade plate, developed with *n*-hexane – dichloromethane – methanol – water (40:50:10:1, *V*/*V*/*V*/*V*) up to 70 mm and detected at white light illumination (a), UV 254 nm (b), and FLD 366 nm (c).

Table S1. List of the ten investigated tomato products extracted with 5 mL *n*-butanol and filtered through 0.45 µm cellulose acetate filter.

No.	Product name	German Manufacturer	LOT no.	*W* [g]	Ingredients	Product appearance
1	Knorr Tomato al Gusto Basilikum	Unilever, Heilbronn	L113102852	5.00	88% tomatoes[a] (65% diced, 23% strained), 4.2% tomato paste[a], 3.5% onion[a], herbs (basil[a], parsley[a], basil powder), sugar iodized table salt, starch, native olive oil, spices (garlic, pepper), acidity regulator: citric acid. [a]from sustainable production.	sauce
2	Maggi rustikale Tomatencremesuppe	Nestlé, Lüdinghausen	12070703U	5.03	30% tomatoes, wheat flour, corn starch, sugar, iodized salt, spices (onion, bell pepper, garlic, pepper), herbs (laurel, majoram), smoked bacon (bacon, smoke), sunflower oil, yeast extract, flavours, thickening agents: guar gum. May contain traces of milk, egg, mustard, soy, and celery.	dry powder
3	Maggi Spaghetti Napoli	Nestlé, Lüdinghausen	121607Q3R	5.02	39.4% tomatoes[b], pea starch[b], potato starch[b], sugar[b], iodized salt (salt, potassium iodate), spices[b] (onoin, garlic, pepper), herbs[b] (parsley, oregano, basil, majoram, thyme), sunflower oil[b], salt[b], seasoning[b] (from wheat), flavours, acidity regulator: citric acid[b]. [b]natural ingredients. May contain traces of milk, egg, celery, mustard, and soy.	dry powder

No.	Product name	German Manufacturer	LOT no.	*W* [g]	Ingredients	Product appearance
4	Kania Tomatensuppe toskanische Art	Radolf Nahrungsmittel, Radolfzell	L1175520658/A	5.01	36% tomatoes, starch, wheat semolina, palm fat, sweet whey powder, sugar, table salt, wheat flour, 2.9% rosted onion (onion, sunflower oil), spices, herbs, yeast extract, acidity regulator: citric acid, beet powder, garlic, dye: beta-carotene, seasoning extract, thickening agents: guar gum. Celery. (May contain traces of egg and soy.)	dry powder
5	Heinz Cream of Tomato Soup	KraftHeinz Company, Düsseldorf	Not available	5.26	89% tomatoes, water, modified starch, sugar, rapeseed oil, skim milk powder, table salt, cream (milk), whey products (milk), acidity regulator: citric acid, seasoning extracts, herbal extracts.	soup
6	Campo Verde Tomaten Sauce Classico	Campo Verde, Uhldingen-Mühlhofen	1163 ST K8	5.09	94.2% tomatoes, 2% native olive oil, 2% basil, 1.2% onion, grape juice, sea salt	sauce
7	Bertolli Siciliana	Unilever, Heilbronn	L11820086	5.11	76% tomatoes, 11% tomato paste, sunflower oil, white wine, onion, carrot, white wine vinegar, garlic, 0,9% dried tomatoes, table salt, sugar, native olive oil, oregano, thyme, basil, rosemary, laurel leaves, acidity regulator: citric acid, chili.	sauce

No.	Product name	German Manufacturer	LOT no.	*W* [g]	Ingredients	Product appearance
8	Ja! Tomaten-Creme-Suppe	Buss Fertiggerichte, Ottersberg	TCSV 3	5.06	48% tomato paste, water, 14% tomatoes, 5% cream, sugar, table salt, rapeseed oil, modified starch, thickening agents: guar gum, xanthan gum, wheat flour, coloring food bell pepper extract, chicken egg white, skim milk powder, spices (containing celery, mustard).	soup
9	REWE Tomatencremsupe	Buss Fertiggerichte, Ottersberg	TCR 3	5.10	56% tomato, water, 5% cream, sugar, table salt, rapeseed oil, modified starch, herbs, thickening agents: guar gum, spices (containing mustard, celery), antioxidants: ascorbic acid, wheat flour, skim milk powder, chicken egg white	soup
10	Self-made tomato soup	Tamara Schreiner, Giessen		5.04	8 tomatoes (skinned,sliced), 1 onion (sautéed), 1 garlic clove (sautéed), 1 soup spoon olive oil, thyme, dried, 6 leaves of basil, 1 teaspoon walnut oil, salt, pepper	soup

Table S2. Investigation of mobile phase systems for tomato extract separation on HPTLC silica gel 60 F_{254} MS-grade plates up to a migration distance of 75 mm (*70 mm).

No.	Mobile Phase (*V*/*V*/*V*/*V*/*V*)
1	*n*-hexane – ethyl acetate – methanol (6:3:2)
2	*n*-hexane – ethyl acetate – methanol (6:3:1)
3	*n*-hexane – toluene – ethyl acetate – methanol – formic acid (20:20:15:5:3)
5	*n*-hexane – toluene – ethyl acetate – methanol – formic acid (20:20:15:5:1)
5*	ethyl acetate – methanol – water (80:13:7)
6*	ethyl acetate – methanol – water (75:17:8)
7*	toluene – ethyl acetate – formic acid (45:30:2)
8*	toluene – acetonitrile – formic acid (45:30:2)
9	toluene – ethyl acetate – formic acid (45:40:2)
10*	*n*-hexane – toluene – ethyl acetate – methanol (4:4:3:2)
11*	ethyl acetate – methanol – water (16:2:1)
12	ethyl acetate – toluene – methanol – water (16:4:2:1)
13*	diisopropyl ether – *n*-butanol – methanol (1:1:1)
14*	ethyl acetate – toluene – methanol – water (16:2:2:1)
15	*n*-hexane – dichloromethane – methanol – water (40:50:12:1)
16	*n*-hexane – dichloromethane – methanol – water (40:50:10:1)
17	*n*-hexane – dichloromethane – methanol – water (40:55:12:1)
18	*n*-hexane – dichloromethane – methanol – water (40:60:15:1)
19	*n*-hexane – dichloromethane – methanol – water (50:55:15:1)
20	*n*-hexane – dichloromethane – methanol – water (50:60:15:1)
21	*n*-hexane – dichloromethane – methanol – acetic acid (40:50:10:1)
22	*n*-hexane – dichloromethane – methanol – acetic acid (80:100:20:1)
23	*n*-hexane – dichloromethane – methanol – water (90:100:24:1)
24	*n*-hexane – dichloromethane – methanol – water (30:50:7:1)

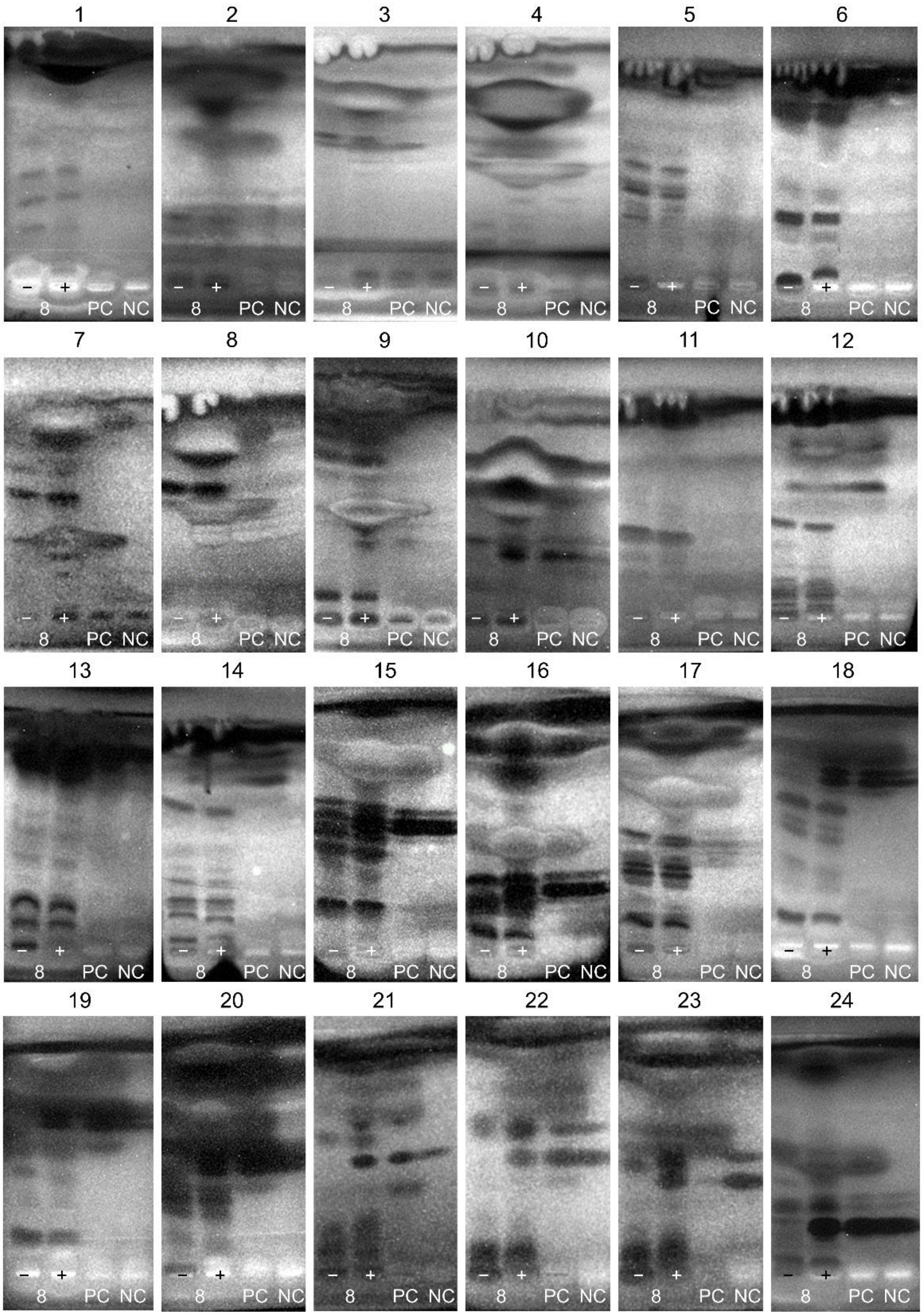

Fig. S1. Investigation of mobile phase systems (no. 1–24, Table S2) on HPTLC plates silica gel 60 F_{254} MS-grade for the NP-HPTLC−nanoGIT$^{+active}$–*Aliivibrio fischeri* separation of the raw (−) *versus* panc-digested (+) extract of tomato product no. 8 (Table S1) along with positive control (PC, rapeseed oil) and negative control (NC, enzyme mix and bile salts) applied (all 5 µL/band each), developed and detected via the bioluminescence (depicted as greyscale image) after the *A. fischeri* bioassay.

a

flow [mL/min]	time [min]	%A	%B
0.3	0	98	2
0.3	2	98	2
0.3	4.5	80	20
0.3	8	10	90
0.3	10	10	90
0.3	10.1	98	2
0.3	13	98	2

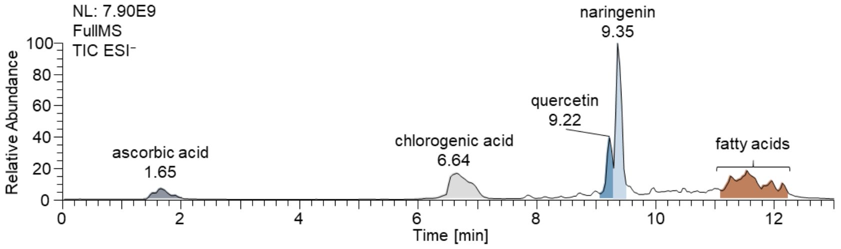

b

flow [mL/min]	time [min]	%A	%B
0.3	0	98	2
0.3	2	98	2
0.3	3.5	80	20
0.3	6	0	100
0.3	8	0	100
0.3	8.1	98	2
0.3	10	98	2

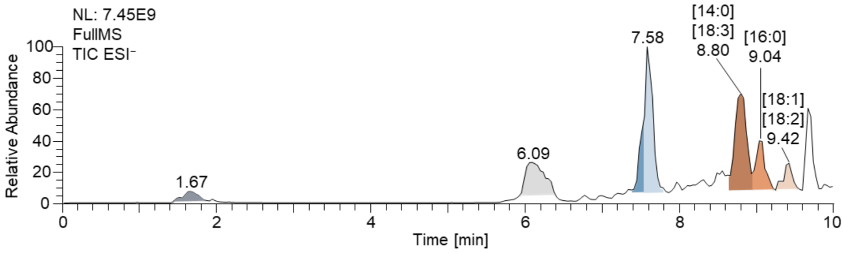

c

flow [mL/min]	time [min]	%A	%B
0.3	0	98	2
0.3	2	98	2
0.3	7	0	100
0.3	9	0	100
0.3	9.1	98	2
0.3	11	98	2

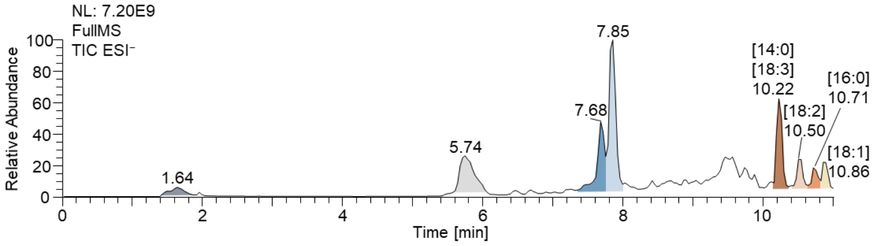

d

flow [mL/min]	time [min]	%A	%B
0.4	0	98	2
0.4	2	98	2
0.4	7	0	100
0.4	10	0	100
0.4	10.1	98	2
0.4	12	98	2

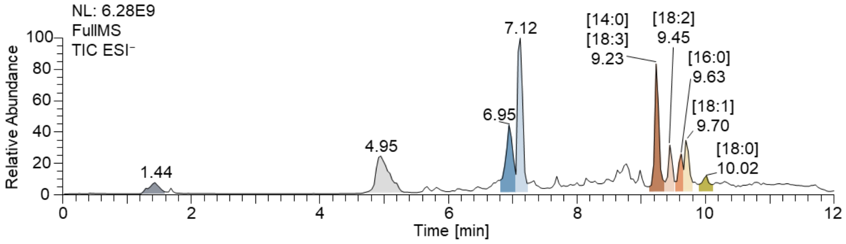

Fig. S2. Development of the RP-HPLC gradient consisting of eluent A (2.5 mM ammonium acetate pH adusted with acetic acid to 4.5) and eluent B (methanol) using standard mixture (500 ng/area, 4 mm × 2 mm) applied on HPTLC silica gel 60 F_{254} MS-grade plate and heart-cut eluted with water – methanol (9:1 *V*/*V*) to the column and HRMS showing the total ion current (TIC) chromatogram in the negative ionization mode.

HPLC Gradient

Properties Gradient		
**General**		
Runtime	0 to 10 min	
Flow	0.3 mL/min	
**Eluents**		
A	4 mM ammonium formate + 0.1% FA in water	
B	4 mM ammonium formate + 0.1% FA in methanol	
**Gradient composition**		
**Time [min]**	**%A**	**%B**
0.0	95	5
1.0	95	5
2.0	50	50
6.0	5	95
8.0	5	95
8.1	95	5
10.0	95	5

Full Scan – AIF

Properties Full Scan	
**General**	
Runtime	0 to 10 min
Polarity	switching (+/−)
**Full MS**	
Resolution	70.000
AGC target	3e6
Scan range	100 to 1000 *m/z*
**Properties AIF**	
**General**	
Runtime	0 to 10 min
Polarity	switching (+/−)
**AIF**	
Resolution	70.000
AGC target	3e6
(N)CE/stepped (N)CE	20, 40, 60
Scan range	100 to 1000 *m/z*
**Lock masses**	
301.14103	positive
413.26623	positive
112.98563	negative

Full Scan – ddMS2

Properties Full Scan	
**General**	
Runtime	0 to 10 min
Polarity	switching (+/−)
**Full MS**	
Resolution	70.000
AGC target	3e6
Scan range	100 to 1000 *m/z*
**Properties ddMS2**	
**General**	
Runtime	0 to 10 min
Polarity	switching (+/−)
**AIF**	
Resolution	17.500
AGC target	3e6
Loop count	5
MSX count	1
TopN	5
Isolation window	4.0 *m/z*
(N)CE/stepped (N)CE	20, 40, 60
Scan range	100 to 1000 *m/z*
**Lock masses**	
301.14103	positive
413.26623	positive
112.98563	negative

Full Scan – mDIA

Properties Full Scan		
**General**		
Runtime	0 to 10 min	
Polarity	negative	
**Full MS – SIM**		
Resolution	70.000	
AGC target	3e6	
Scan range	100 to 1000 *m/z*	
**Properties mDIA**		
**General**		
Runtime	0 to 10 min	
Polarity	negative	
**mDIA**		
Resolution	35.000	
AGC target	3e6	
Loop count	18	
MSX count	6	
Isolation window	50.0 *m/z*	
(N)CE/stepped (N)CE	20, 40, 60	
Scan range	100 to 1000 *m/z*	
**Lock masses**		
112.98563	negative	
**Inclusion list**		
**Mass [*m/z*]**	**polarity**	**MSX ID**
125.00000	negative	1
175.00000	negative	2
225.00000	negative	3
275.00000	negative	4
325.00000	negative	5
375.00000	negative	6
425.00000	negative	7
475.00000	negative	8
525.00000	negative	9
575.00000	negative	9
625.00000	negative	10
675.00000	negative	10
725.00000	negative	11
775.00000	negative	11
825.00000	negative	11
875.00000	negative	11
925.00000	negative	11
975.00000	negative	11

Full Scan – vDIA

Properties Full Scan	
**General**	
Runtime	0 to 10 min
Polarity	negative
**Full MS – SIM**	
Resolution	70.000
AGC target	3e6
Scan range	100 to 1000 *m/z*
**Properties vDIA**	
**General**	
Runtime	0 to 10 min
Polarity	negative
**vDIA (1)**	
Resolution	35.000
AGC target	3e6
Loop count	8
MSX count	1
Isolation window	50.0 *m/z*
(N)CE/stepped (N)CE	20, 40, 60
Scan range	100 to 1000 *m/z*
**vDIA (2)**	
Resolution	35.000
AGC target	3e6
Loop count	2
MSX count	1
Isolation window	100.0 *m/z*
(N)CE/stepped (N)CE	20, 40, 60
Scan range	100 to 1000 *m/z*
**vDIA (3)**	
Resolution	35.000
AGC target	3e6
Loop count	1
MSX count	1
Isolation window	300.0 *m/z*
(N)CE/stepped (N)CE	20, 40, 60
Scan range	100 to 1000 *m/z*
**Lock masses**	
112.98563	negative
**Inclusion list**	
**Mass [*m/z*]**	**polarity**
125.00000	negative
175.00000	negative
225.00000	negative
275.00000	negative
325.00000	negative
375.00000	negative
425.00000	negative
475.00000	negative
550.00000	negative
650.00000	negative
850.00000	negative

Fig. S3. RP-HPLC–HESI-HRMS/MS settings for the evaluation of a non-target acquisition strategy. Full scan settings were maintained while MS2 settings were varied as all ion fragmentation (AIF), data-dependent MS2 (ddMS2), multiplexed data-independent acqusition (mDIA), and variable data-independet acquisition (vDIA).

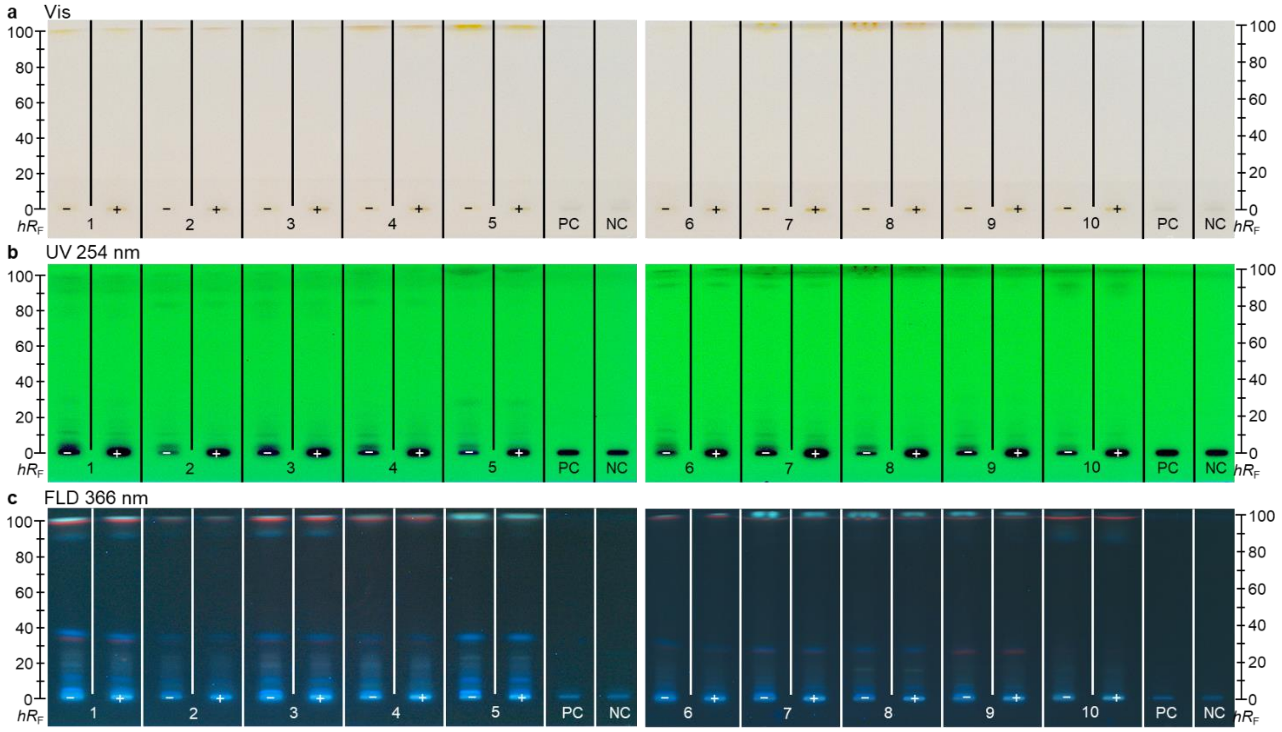

Fig. S4. NP-HPTLC−nanoGIT$^{+active}$–UV/Vis/FLD profiles of 10 raw (−) *versus* panc-digested (+) tomato products (5 µL/band, Table S1) along with positive control (PC, rapeseed oil), and negative control (NC, pancreatic enzyme mix and bile salts) on HPTLC silica gel 60 F_{254} MS-grade plate, developed with *n*-hexane – dichloromethane – methanol – water (40:50:10:1, *V*/*V*/*V*/*V*) up to 70 mm and detected at white light illumination (a), UV 254 nm (b), and FLD 366 nm (c).
